# Estimating lung cancer risk from chest X-ray and symptoms: a prospective cohort study

**DOI:** 10.3399/bjgp20X713993

**Published:** 2020-12-15

**Authors:** Stephen H Bradley, Nathaniel Luke Fielding Hatton, Rehima Aslam, Bobby Bhartia, Matthew EJ Callister, Martyn PT Kennedy, Luke TA Mounce, Bethany Shinkins, William T Hamilton, Richard D Neal

**Affiliations:** Leeds Institute of Health Sciences, University of Leeds;; Leeds Teaching Hospitals NHS Trust.; Calderdale and Huddersfield NHS Trust.; Leeds Teaching Hospitals NHS Trust.; Leeds Teaching Hospitals NHS Trust.; Leeds Teaching Hospitals NHS Trust.; College of Medicine & Health, University of Exeter.; Test Evaluation Group, Leeds Institute of Health Sciences, University of Leeds.; College of Medicine & Health, University of Exeter.; Leeds Institute of Health Sciences, University of Leeds;

**Keywords:** chest x-ray, early diagnosis, general practice, lung cancer, primary care, radiograph

## Abstract

**Background:**

Chest X-ray (CXR) is the first-line investigation for lung cancer in many countries but previous research has suggested that the disease is not detected by CXR in approximately 20% of patients. The risk of lung cancer, with particular symptoms, following a negative CXR is not known.

**Aim:**

To establish the sensitivity and specificity of CXR requested by patients who are symptomatic; determine the positive predictive values (PPVs) of each presenting symptom of lung cancer following a negative CXR; and determine whether symptoms associated with lung cancer are different in those who had a positive CXR result compared with those who had a negative CXR result.

**Design and setting:**

A prospective cohort study was conducted in Leeds, UK, based on routinely collected data from a service that allowed patients with symptoms of lung cancer to request CXR.

**Method:**

Symptom data were combined with a diagnostic category (positive or negative) for each CXR, and the sensitivity and specificity of CXR for lung cancer were calculated. The PPV of lung cancer associated with each symptom or combination of symptoms was estimated for those patients with a negative CXR.

**Results:**

In total, 114 (1.3%) of 8996 patients who requested a CXR were diagnosed with lung cancer within 1 year. Sensitivity was 75.4% and specificity was 90.2%. The PPV of all symptoms for a diagnosis of lung cancer within 1 year of CXR was <1% for all individual symptoms except for haemoptysis, which had a PPV of 2.9%. PPVs for a diagnosis of lung cancer within 2 years of CXR was <1.5% for all single symptoms except for haemoptysis, which had a PPV of 3.9%.

**Conclusion:**

CXR has limited sensitivity; however, in a population with a low prevalence of lung cancer, its high specificity and negative predictive value means that lung cancer is very unlikely to be present following a negative result. Findings also support guidance that unexplained haemoptysis warrants urgent referral, regardless of CXR result.

## INTRODUCTION

In many healthcare systems, chest X-ray (CXR) remains the first-line test for lung cancer for patients who are symptomatic. The relatively poor survival rates of people with lung cancer in countries such as the UK, compared with other countries, may be partly due to the disease being diagnosed in its later stages.^1^^,^^2^ More evidence on the accuracy of CXR to determine whether it has sufficient sensitivity and specificity for lung cancer, and whether certain symptoms confer a greater risk that warrants immediate referral for alternative investigations, even following a negative CXR, is required.

In the UK, guidance from the National Institute for Health and Care Excellence (NICE) on lung cancer diagnosis^3^ draws on the positive predictive values (PPVs) of symptoms, which have been derived from cohort and case-control studies.^4^^–^^6^ NICE guidance recommends CXR for several symptoms in patients aged >40 years.^3^ In turn, findings on CXR that are suspicious of lung cancer warrant an urgent referral, which means the individual should be seen by a specialist within 2 weeks; this is known as a 2-week wait or TWW referral. As a result of its relatively high PPV — reported at 2.4% by William Hamilton^4^ — the presence of unexplained haemoptysis is considered justification to initiate a TWW referral irrespective of CXR results.

Although guidance for GPs is clear on when to consider a CXR and the action to take if an X-ray is abnormal, what GPs should do when CXR does not indicate grounds for TWW referral is less clear. A recent systematic review^7^ estimated that CXR detects lung cancer in approximately 77%–80% of cases in the year before diagnosis; in addition, audits^8^^,^^9^ have suggested that negative CXR results in patients later diagnosed with lung cancer may contribute to a delay in diagnosis. The study presented here utilised routinely collected data for patients aged >50 years who presented to a service that allowed them to request a CXR (a self-requested CXR, or SR-CXR) if they had any symptom that, according to NICE,^3^ warrants CXR.

The study aimed to:

- determine the sensitivity and specificity of CXR for lung cancer in patients with symptoms aged >50 years, who requested the investigation;
- estimate the risk (PPVs) of being diagnosed with lung cancer within 1 year and 2 years following a negative CXR result for a range of symptoms and symptom combinations; and
- determine whether the symptoms associated with lung cancer are different in those who had a positive CXR result compared with those who had a negative CXR result.

**Table:** How this fits in

Chest X-ray (CXR) is the first-line test for lung cancer in many countries. Some referral guidelines recommend CXR for individuals who have particular symptoms, based on those symptoms’ positive predictive values for lung cancer. It is known that CXR does not identify lung cancer in around a fifth of cases, but the risk of different symptoms being predictive of lung cancer in the context of a negative CXR is not known. This study, which was based on a service that allowed patients to request a CXR if they had symptoms of lung cancer, suggests the risk of being diagnosed with the disease following a negative CXR is very low, except in patients with haemoptysis.

## METHOD

### Study design

In this prospective cohort study, the authors utilised routinely collected data that had been obtained between January 2011 and October 2016 from an SR-CXR service at Leeds Teaching Hospitals NHS Trust (LTHT), England. LTHT is a large trust, with radiology and lung cancer services, which serves a population of approximately 780 000.^10^

The SR-CXR service was set up as a component of an early-diagnosis campaign for lung cancer in Leeds, which was initially funded by the National Awareness and Early Diagnosis Initiative. The service allowed patients aged >50 years who had symptoms warranting investigation with CXR, as outlined in the NICE guidance, to have this investigation without requiring a GP referral.^11^ The relevant symptoms were cough, haemoptysis, dyspnoea, chest pain, weight loss, and change in voice. Patients who had received chest radiography in the previous three months were not eligible for SR-CXR. The data that were routinely collected included symptoms prompting CXR and smoking status, as well as the resulting CXR report.

Patients who had a history of lung cancer prior to attending for SR-CXR were excluded, as were those who were diagnosed with an intrathoracic malignancy other than lung cancer in the 2 years following SR-CXR.

The written reports of all SR-CXRs were coded as positive or negative, based on the criteria used in a Royal College of Radiologists national audit.^12^ These are summarised in Supplementary Table S1. To confirm satisfactory inter-reviewer reliability using this coding system, two authors independently coded a sample of 100 CXR reports; this yielded a Cohen’s κ score of 0.92, indicating a high level of agreement between reviewers.

A study database was created using symptom and smoking data from the SR-CXR service, which was supplemented with the codes allocated for each CXR report. For patients who underwent >1 SR-CXR during the study, each SR-CXR was considered a separate event. Demographic data were obtained from LTHT’s electronic patient records. In order to reflect NICE guidance,^3^ patients who had thrombocytosis (defined as a platelet count of >400 x 10^9^/L) were identified if they had a full blood count (FBC) in the 1 year or 2 years prior to SR-CXR from the LTHT’s pathology system.

By cross-referencing with a database of all patients who received a multidisciplinary team-approved diagnosis of lung cancer in LTHT between 2011 and 2018, patients who were diagnosed with lung cancer between 1 year and 2 years following SR-CXR were identified. The presence or absence of a diagnosis of lung cancer on the database within 1 year and 2 years following SR-CXR was used to determine whether a diagnosis of lung cancer had occurred.

### Statistical analysis

The incidences of patients diagnosed with lung cancer within 1 year and 2 years following a negative CXR were calculated, followed by the incidences of lung cancer within 1 year and 2 years by each symptom and symptom combination; these are equivalent to the observed PPVs of each symptom or symptom combination. Patients who reported multiple symptoms were included in calculations for individual symptoms and also for symptom combinations.

Estimates of PPVs adjusted for age, sex, and smoking status were obtained from the marginal distributions of separate logistic regression models predicting lung cancer diagnosis within 1 year of a negative CXR for each symptom and symptom combination. Adjusted PPVs were derived for diagnosis within 2 years using the same method. Comparing the average risk for people with a symptom with the average risk for people without that symptom allowed a percentage estimate of the additional risk of cancer for those with each symptom or symptom combination to be derived. All patients in the study population had symptoms, so non-smoking, female patients aged 50–55 years were used as a reference category as they had the lowest risk of lung cancer.

A further logistic regression model predicting lung cancer diagnosis within 2 years was constructed for each symptom and symptom combination; this included the interaction between the indicator for that symptom/symptom combination and CXR result, as well as the main effects for both variables. These interactions explored whether the association of each symptom and symptom combination with lung cancer diagnosis differed between patients with a positive CXR and those with a negative CXR.

Statistical analysis was undertaken using IBM SPSS Statistics (version 25).

## RESULTS

In total, 9367 SR-CXRs were performed during the 70-month study period. Of all records, 342 were excluded due to errors in the completion of patient information at the time of attending for SR-CXR, which meant that patient and CXR records could not be identified. An additional 16 patients were excluded because they had had lung cancer diagnosed prior to attending for SR-CXR, and a further 13 were excluded because they were subsequently diagnosed with intrathoracic malignancies other than lung cancer. The majority of these excluded cases were mesothelioma; however, due to data-suppression requirements to maintain the anonymity of patients, it was not possible to enumerate individual malignancies. Following the stated exclusions, the study population was 8996; characteristics of the study population are outlined in Table 1.

**Table 1.** Patient characteristics, N = 8996

**Characteristic**	***n* (%)**
**Sex**	
Male	4441 (49.4)
Female	4555 (50.6)

**Smoking status^a^**	
Smoker status not recorded	60 (0.7)
Smoker/ex-smoker	5951 (66.2)
Never smoked	2985 (33.2)

**Age group, years**	
50–55	1485 (16.5)
56–60	1484 (16.5)
61–65	1777 (19.8)
66–70	1598 (17.8)
71–75	1205 (13.4)
76–80	845 (9.4)
>80	602 (6.7)

**Symptoms recorded, *n***	
1	3527 (39.2)
2	3240 (36.0)
3	1674 (18.6)
≥4	555 (6.2)

**Thrombocytosis**	
Thrombocytosis in 24 months prior to SR-CXR^b^	395 (7.2)

**Index of Multiple Deprivation score, decile**	
1 (greatest deprivation)	2844 (31.6)
2	2233 (24.8)
3	1350 (15.0)
4	1652 (18.4)
5	753 (8.4)
6	29 (0.3)
7	38 (0.4)
8	37 (0.4)
9	35 (0.4)
10 (least deprivation)	25 (0.3)

a Category in which stated percentages do not total 100 due to rounding.

b Out of 5524 people, who had FBC. FBC = full blood count (the blood test routinely used to detect thrombocytosis). SR-CXR = self-requested chest X-ray.

A total of 114 patients (1.3%) were diagnosed with lung cancer within 1 year of having the SR-CXR, of whom 86 (75.4%) had a positive SR-CXR result; the remaining 28 (24.6%) had a negative SR-CXR result (Table 2). Negative predictive value for a diagnosis of lung cancer within 1 year was 99.7%. At 2 years following SR-CXR, a total of 154 patients (1.7%) were diagnosed with lung cancer, of whom 97 (63.0%) had a positive SR-CXR result; the remaining 57 (37.0%) had a negative SR-CXR result. Demographic and health characteristics of those diagnosed with lung cancer are given in Table 2; Table 3 summarises the test characteristics of SR-CXRs undertaken in the study population.

**Table 2.** Characteristics of patients diagnosed with lung cancer within 1 year and 2 years of SR-CXR

**Characteristic**	**Diagnosis in 1 year following SR-CXR**	**Diagnosis in 2 years following SR-CXR**
**Patients, *n* (%)**	114 (1.3)	154 (1.7)

**Mean age, years**	69	70

**Male, *n* (%)**	45 (39.5)	64 (41.6)

**Smoker/ex-smoker, *n* (%)**	107 (93.9)	145 (94.2)

**SR-CXR result, *n* (%)**		
Positive	86 (75.4)	97 (63.0)
Negative	28 (24.6)	57 (37.0)

**Stage at diagnosis, *n* (%)**		
Stage I–II	34 (29.8)	50 (32.5)
Stage III–IV	80 (70.2)	104 (67.5)

**Tumour histology, *n* (%)**		
Adenocarcinoma	38 (33.3)	50 (32.5)
Squamous cell carcinoma	31 (27.2)	41 (26.6)
Small cell carcinoma	15 (13.2)	22 (14.3)
Non-small cell carcinoma not otherwise stated and large cell^a^	17 (14.9)	18 (11.7)
Unknown	13 (11.4)	23 (14.9)

a *Non-small cell group carcinoma not otherwise stated and large cell group data have been combined due to data suppression requirements that prevent reporting of identifiable groups of* <*5. SR-CXR = self-requested chest X-ray.*

**Table 3.** Test characteristics of SR-CXR in the study population

**Test characteristic**	**Lung cancer diagnosis (within 1 year of SR-CXR)**	**No diagnosis of lung cancer (within 1 year of SR-CXR)**	**Total, *n***
Positive x-ray result, *n*	86	867	953
Negative x-ray result, *n*	28	8015	8043
Total, *n*	114	8882	8996
Sensitivity, % (95% CI)	75.4 (67.5 to 83.3)	—	—
Specificity, % (95% CI)	90.2 (89.6 to 90.9)	—	—
PPV, % (95% CI)	9.02 (7.21 to 10.8)	—	—
NPV, % (95% CI)	99.7 (99.5 to 99.8)	—	—

NPV = negative predictive value. PPV = positive predictive value. SR-CXR = self-requested chest X-ray.

**Figure 1.**
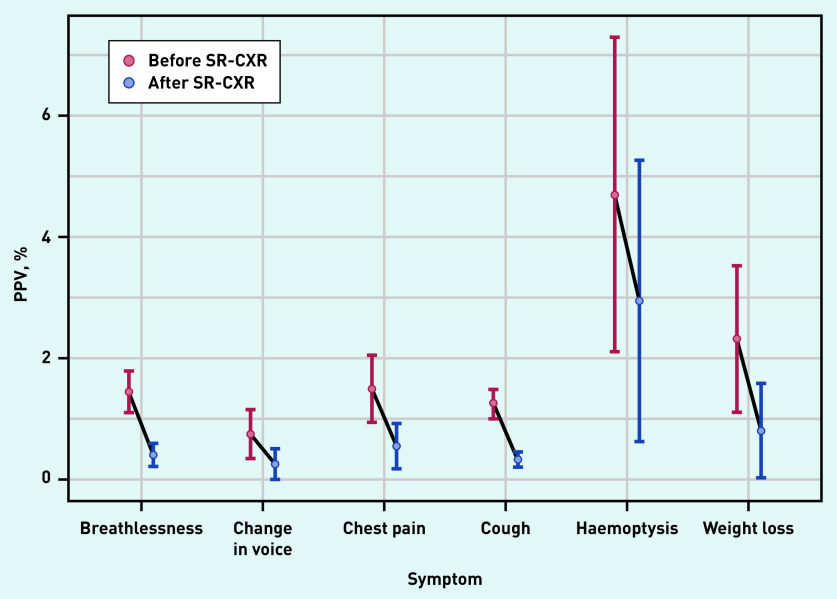
**Individual symptoms’ PPVs (unadjusted) of lung cancer diagnosis in 1 year following SR-CXR. As thrombocytosis was not used as a qualifying symptom for chest x-ray, it has not been included. Error bars indicate 95% confidence intervals. PPV = positive predictive value. SR-CXR = self-requested chest X-ray.**

**Figure 2.**
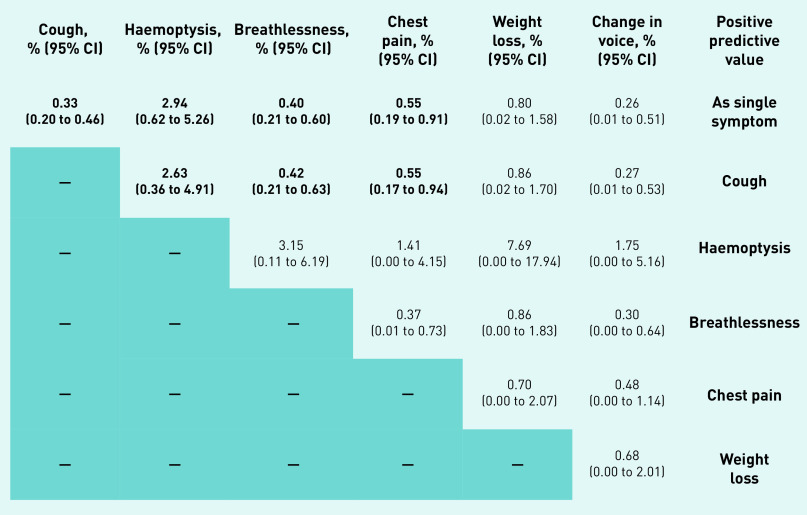
***Positive predictive values for lung cancer diagnosis of symptoms^a^ and symptom combinations at 1 year following SR-CXR in those with a negative SR-CXR result. ^a^As thrombocytosis was not used as a qualifying symptom for chest X-ray, it has not been included. Unbolded data from <5 cases. SR-CXR = self-requested chest X-ray.***

Observed cancer incidence for patients with a negative SR-CXR for 1 year and 2 years following SR-CXR was 0.35% (95% CI = 0.22 to 0.48) and 0.71% (95% CI = 0.53 to 0.89), respectively (Tables 1 and 2). One-year and 2-year incidences adjusted for age, sex, and smoking status obtained from the logistic regression model were 0.27% (95% CI = 0.13 to 0.55) and 0.56% (95% CI = 0.37 to 0.84), respectively (data not shown). Figure 1 contains the observed PPVs of lung cancer in the 1-year period after SR-CXR for single symptoms in the whole study population and those participants with a negative SR-CXR result. Figure 2 contains the observed PPVs of single symptoms and symptom combinations in those who had a diagnosis of lung cancer within 1 year of a negative SR-CXR result.

**Figure 3.**
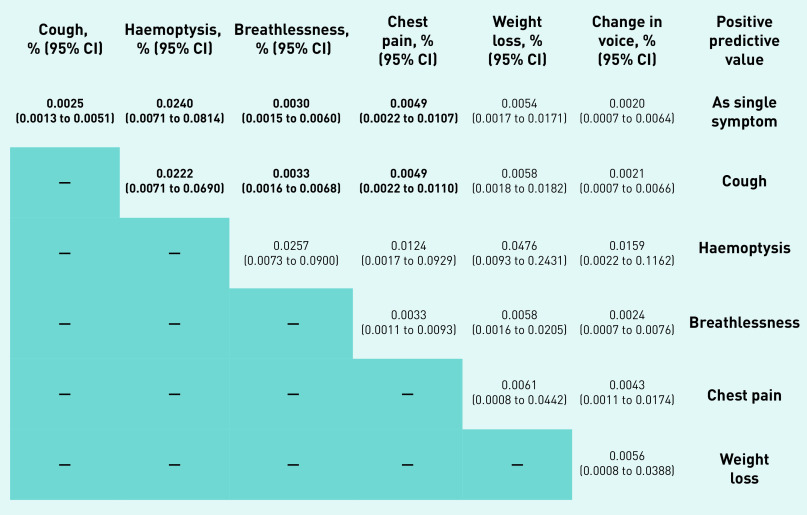
***Adjusted symptom combination marginal means^a^ for lung cancer in the 1-year period following negative SR-CXR.^a^Marginal means are the average risks of cancer for different groups (for example, those with or without a symptom), taking into account the characteristics (such as age and sex) of the people in each group. When the average risk for people with a symptom is compared with that for people without that symptom, it gives an estimate of the additional risk of cancer for people with the symptom. Unbolded data from <5 cases. SR-CXR = self-requested chest X-ray.***

Figure 3 contains the adjusted marginal mean for single symptoms and symptom combinations within 1 year following a negative SR-CXR.

The 1-year and 2-year observed PPVs of symptom combinations for the entire study population, as well as the 2-year adjusted and observed PPVs for those who had a negative SR-CXR, are detailed in Supplementary Figures S1–S4.

In total, 4135 and 5524 patients had FBCs obtained in the 1 year and 2 years prior to SR-CXR, respectively; of these, 217 and 395 had thrombocytosis. In all analyses of thrombocytosis with other symptoms, the lower 95% CIs for PPVs were all ≤0 or could not be calculated due to insufficient cases; as such, inclusion of thrombocytosis did not add any discriminative utility in this study. The 1-year PPVs for the entire study population for thrombocytosis were 1.03 (95% CI = 0.00 to 2.45) when in combination with cough, 2.17 (95% CI = 0.00 to 6.39) in combination with chest pain, and 6.67 (95% CI = 0.00 to 15.59) in combination with weight loss (data not shown). There was no evidence that symptoms’ associated risk of lung cancer differed according to the result of the SR-CXR (all interaction *P*-values >0.05).

Table 3 reports the diagnostic accuracy estimates of SR-CXR.

## DISCUSSION

### Summary

This study presents the PPVs for developing lung cancer with respect to particular symptoms when reported in a service that allowed patients to have an SR-CXR. The findings suggested that, for most symptoms, the risk of being diagnosed with lung cancer following a negative CXR remains very low; the exception was haemoptysis, which had a PPV of 2.9%. This provides evidence to support current NICE guidance, which considers an abnormal CXR result as the main criterion for a TWW referral; the exception is haemoptysis, which warrants referral, even in the absence of a CXR.^3^ We found no evidence that different symptoms were associated with a diagnosis of lung cancer in those with a negative CXR result compared to those who had a positive CXR.

The sensitivity of CXR for a diagnosis of lung cancer at 1 year was 75.4%; however, coupled with a negative predictive value (NPV) of 99%, CXR could be considered well suited to its role as a first-line investigation in a low-prevalence setting.

### Strengths and limitations

The large sample size of the study population is a strength of this study, along with its prospective design. However, the study population had a low prevalence of lung cancer with only 154 (1.7%) diagnosed with the disease within 2 years, of whom 57 (37.0%) had had a negative SR-CXR result. This meant that insufficient cases were present to calculate PPVs for several symptom combinations. In addition, the lack of a control group meant the calculation of adjusted PPVs was calculated using a within-study comparator based on the patients at lowest risk of developing lung cancer.

In determining which study participants developed the disease, the authors assumed that patients did not move outside of the region after having their SR-CXR and that diagnoses were recorded in LTHT. It is possible that this approach underestimated the prevalence of lung cancer if some patients moved and were subsequently diagnosed elsewhere.

It is also possible that some cancers diagnosed within 1 year and 2 years following SR-CXR were not actually present at the time of imaging. Although the natural history of undetected lung cancer is necessarily unknown, estimates derived from screening studies suggest that, in a large proportion of cases, lung cancer develops over some years prior to detection.^13^^–^^15^ However, a small proportion of cancers develop more rapidly;^16^ therefore, the assumption that a lung cancer that was not detected on SR-CXR constitutes a ‘false negative’ result requires some qualification, particularly at the 2-year interval.

The study population had a CXR at their own request. There could be differences between the study population and the patient population who are referred for CXR by a GP. It is possible that the prevalence of lung cancer in the study population is lower than in patients who report their symptoms to a GP and are then referred for a CXR. If this is the case, the pre-test probability of lung cancer would be lower and result in lower PPVs and sensitivity, and higher NPVs and specificity, than in the referred population. In addition, although people in the study population were deemed eligible for CXR because they had symptoms listed in current NICE guidance, it is important to acknowledge that this guidance was based on GP appraisal of patient symptoms and did not envisage patient-requested investigations. The study population was also limited to individuals aged >50 years, while NICE guidelines suggests investigation for those with symptoms aged >40 years.^3^

### Comparison with existing literature

This study has confirmed the finding of previous studies that haemoptysis is the symptom most strongly associated with a subsequent diagnosis of lung cancer,^4^^–^^6^ and also suggests that haemoptysis remains an important symptom, even following a negative CXR.

Hamilton^4^ undertook a case–control study that linked cancer registry data to GPs’ paper and electronic health record symptom records; that study did not report CXR results. Symptom PPVs for the population of the study presented here were much higher than those reported by Hamilton, although it is not known whether the populations are directly comparable; patients’ decisions to self-request a CXR may reflect a greater underlying concern that serious disease is present, compared with those presenting to a GP with symptoms prior to a decision having been made about investigation with CXR, or those who mention symptoms to a GP while attending for other reasons.

Previous findings that thrombocytosis is associated with an increased risk of lung cancer^17^ were not replicated in this study. The relatively small numbers of patients diagnosed with lung cancer and the number of those with thrombocytosis is likely to have rendered this study underpowered to detect such an association. Another possibility is that the population selected for a FBC and the population that requested a CXR in this study — that is, those selected for testing by their GP or those who opted to undergo a CXR themselves — were at already-elevated risk of serious disease compared with the wider population.^18^ This related mechanism of test selection bias may have obscured the capacity to differentiate elevated risk of thrombocytosis as well as patient self-selection to undergo CXR.

### Implications for practice

This study provides evidence to support existing guidance that advocates urgent referral for unexplained haemoptysis. The study also suggests that CXR’s present role as a first-line test for lung cancer in symptomatic patients is appropriate.

Clinicians should understand that although a diagnosis of lung cancer is uncommon after a negative CXR, the high NPV of the modality reflects the low prevalence of lung cancer among those who have symptoms that are associated with the disease, since most of these symptoms are common and non-specific. Therefore, it is important to remember that CXR does not detect over a fifth of cases of lung cancer and that diagnosis of lung cancer following a negative CXR, though unlikely, remains possible. This insight supports the use of a safety netting approach even following a negative CXR, by advising patients to represent if their symptoms persist or worsen within a particular timeframe.

Clinicians can, with confidence, inform patients who have not had haemoptysis that a diagnosis of lung cancer following a negative CXR is very unlikely and that the benefits of immediate further investigation are, in most cases, unlikely to justify the harms, costs, and inconvenience.

As the precise level of risk that might be considered acceptable will vary between individuals and clinicians, a shared decision-making approach is prudent when considering what action to take when symptoms continue, despite a negative CXR. Clinicians should remember that, even in patients who appear to be at low risk, a negative CXR does not eliminate the possibility of lung cancer and, in some cases, further investigation should be considered if symptoms persist or evolve.
